# Complete genome sequence data of *Leuconostoc mesenteroides* KNU-2 and *Weissella hellenica* MBEL1842 isolated from kimchi

**DOI:** 10.1016/j.dib.2023.108919

**Published:** 2023-01-18

**Authors:** J.A. Yoon, S.Y. Kwun, E.H. Park, M.D. Kim

**Affiliations:** a Department of Food Biotechnology and Environmental Science, Kangwon National University, Chuncheon 24341, Korea; b Department of Food Science and Biotechnology, Kangwon National University, Chuncheon 24341, Korea; c Institute of Fermentation and Brewing, Kangwon National University, Chuncheon 24341, Korea

**Keywords:** Complete genome, Kimchi, Lactic acid bacteria, *Leuconostoc mesenteroides*, *Weissella hellenica*

## Abstract

Kimchi, a traditional Korean fermented food, contains many lactic acid bacteria. *Leuconostoc mesenteroides* KNU-2 strain with low-temperature tolerance and *Weissella hellenica* MBEL1842 with antibacterial activity were isolated from kimchi. The genomes of *L. mesenteroides* KNU-2 and *W. hellenica* MBEL1842 are composed of one circular chromosomal genome of 1,973,419 bp (37.9% G+C content) and 1,887,056 bp (37.9% G+C content), as well as four and one plasmids, respectively, The sequence data of the strains were deposited in GenBank under the accession numbers CP089782 (*L. mesenteroides* KNU-2) and CP086020 (*W. hellenica* MBEL1842).

**Specifications Table**

**Table:** 

Subject	Genetics: General
Specific subject area	Genomics and Molecular Biology
Type of data	Table and Figure
How the data were acquired	The PacBio RSII platform was used for genome sequencing. PacBio long-reads of *L. mesenteroides* KNU-2 were assembled using RS HGAP(v3.0) and annotated using Prokka (v1.12b), while those of *W. hellenica* MBEL1842 were assembled using FALCON (v.2.1.4) and annotated using Prokka (v1.12b).
Data format	Raw and analyzed
Description of data collection	Genomic DNA of *L. mesenteroides* KNU-2 and *W. hellenica* MBEL1842 was extracted and used.
Data source location	• Institution: Kangwon National University • City/Region: Chuncheon, Kangwon-do • Country: Republic of Korea • Latitude and longitude: 37°87′ N and 127°74′ E
Data accessibility	The draft genome sequence of *L. mesenteroides* KNU-2 was deposited in GenBank under the following Biosample, Bioproject, Sequence Read Archive and GenBank accession number, SAMN10492388 (https://www.ncbi.nlm.nih.gov/biosample/?term=SAMN10492388), PRJNA507406 (https://www.ncbi.nlm.nih.gov/bioproject/PRJNA507406), SRX12232645 (https://www.ncbi.nlm.nih.gov/sra/SRX12232645), and CP089782 (https://www.ncbi.nlm.nih.gov/nuccore/CP089782). That of *W. hellenica* MBEL1842 was deposited under SAMN13968964 (https://www.ncbi.nlm.nih.gov/biosample/?term=SAMN13968964), PRJNA604418 (https://www.ncbi.nlm.nih.gov/bioproject/604418), SRX12745381-SRX12745386 (https://www.ncbi.nlm.nih.gov/sra/?term=SRP342772) and CP086020 (https://www.ncbi.nlm.nih.gov/nuccore/CP086020).

## Value of the Data

• The genome sequence of *L. mesenteroides* KNU-2 and *W. hellenica* MBEL1842 provides fundamental knowledge about genes related to lactic acid bacteria isolated from kimchi.
• The genome data of the strains will be useful for comparative genomic analysis with other lactic acid bacteria.
• The data can be useful in understanding the characterization of enzymes and genes related to kimchi fermentation.

## 1 Data Description

Kimchi is a traditional Korean fermented food consisting of vegetables such as Chinese cabbage, radish, and various ingredients [3]. Lactic acid bacteria (LAB), such as *Leuconostoc, Lactobacillus*, and *Weissella* genera, play an important role in kimchi fermentation [4,9]. Therefore, LAB are considered fermentation starters that produce standardized, high-quality kimchi [9]. *L. mesenteroides* KNU-2 strain with low-temperature tolerance and *W. hellenica* MBEL1842 with antibacterial activity were isolated from kimchi.

The PacBio reads of *L. mesenteroides* KNU-2 and *W. hellenica* MBEL1842 were assembled. Only the L. *mesenteroides* KNU-2 reads were assembled using RS HGAP (v3.0) to make a circular genome [1]. However, *W. hellenica* MBEL1842 reads were then assembled again using Falcon (v2.1.4) to obtain a circular genome [2]. The genome coverage of *L. mesenteroides* KNU-2 was 560 × and that of *W. hellenica* MBEL1842 was 366 ×.

Table 1 summarizes the genomic features of L. *mesenteroides* and *W. hellenica* strains. The genome of L. *mesenteroides* KNU-2 comprises one circular chromosomal genome of 1973,419 bp (37.9% G+C content) and four circular plasmids. The chromosome contains 1957 predicted protein-coding, 12 rRNA, and 71 tRNA genes. The genome of *W. hellenica* MBEL1842 was identified as a 1,887,056 bp (36.9% G+C content) circular chromosome with one circular plasmid. The chromosome has 1,779 predicted protein-coding, 25 rRNA, and 76 tRNA genes. The average nucleotide identity (ANI) value for *L. mesenteroides* KNU-2 and other *L. mesenteroides* strains was over 99%, indicating a high species boundary value (ANI > 95%) (Fig. 2) [8]. The *W. hellenica* MBEL1842 and other *W. hellenica* strains also showed a high ANI value of over 99% (Fig. 3). The whole-genome comparisons of *L. mesenteroides* (Fig. 4) and *W. hellenica* strains (Fig. 5) showed high synteny and no large rearrangements.

**Table 1:** Genomic features of *L. mesenteroides* and *W. hellenica* strains.

Lactic acid bacteria	Strain	Length (bp)	G+C content (%)	Protein-coding genes	rRNA genes	tRNA genes	Plasmid No.	GenBank No.	Refs.
L. *mesenteroides*	KNU-2	1,973,419	37.9	1,957	12	71	4	CP089782	This study
	LK151	2,090,103	37.8	1,974	9	67	3	AP017936	[6]
	ATCC8293	2,038,396	37.7	1,925	12	70	1	CP000414	[10]
	J18	1,900,740	37.8	1,769	12	70	4	CP003101	[5]
	DRC1506	1,893,478	37.7	1,707	12	70	3	CP014611	[7]

*W. hellenica*	MBEL1842	1,887,056	36.9	1,779	25	76	1	CP086020	This study
	0916–4–2	1,875,603	38.9	1,834	25	76	2	CP033608	[12]
	CBA3632	1,900,683	36.8	1,834	25	75	4	CP042399	-

**Fig. 1:**
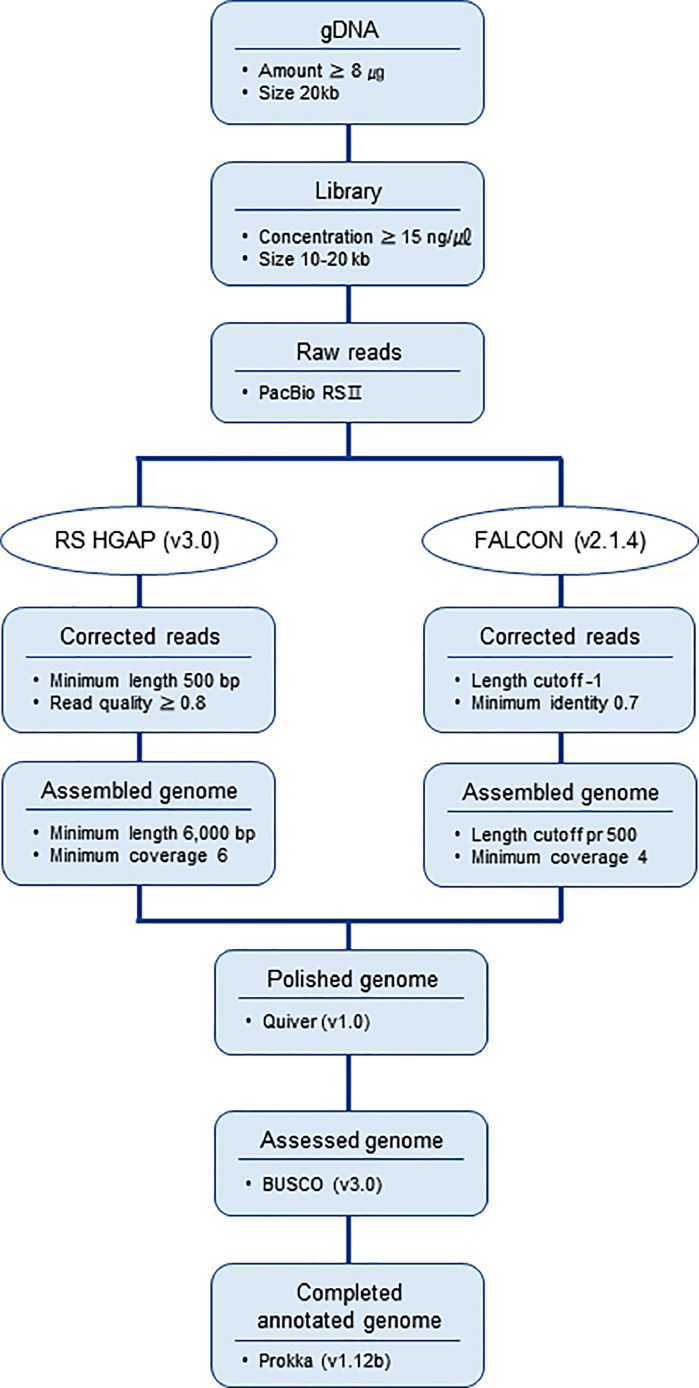
Steps and parameters of whole-genome sequencing.

**Fig. 2:**
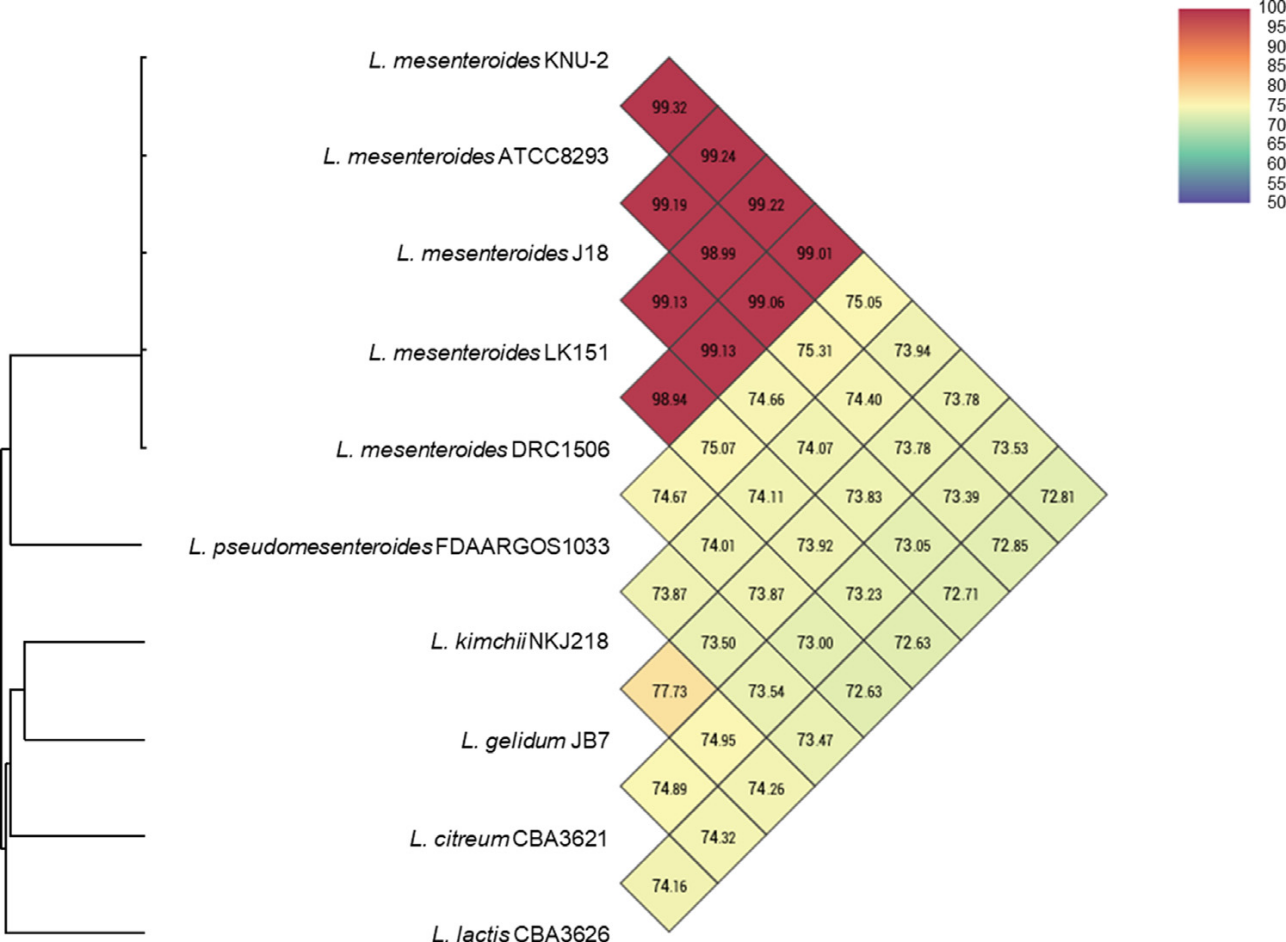
Heatmap generated with Ortho-ANI values between *Leuconostoc mesenteroides* KNU-2 and other closely related species.

**Fig. 3:**
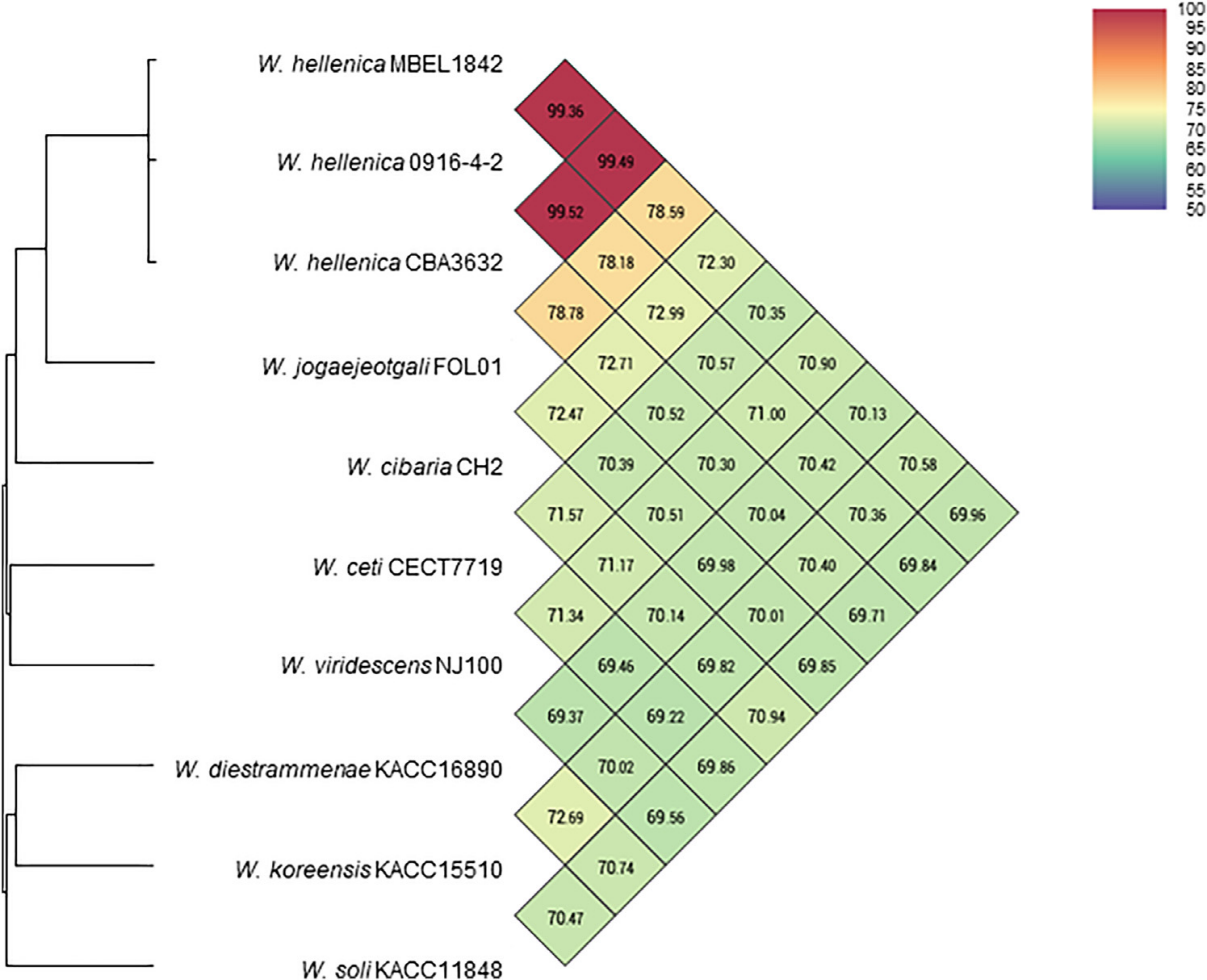
The Heatmap of the Ortho-ANI values of *W. hellenica* MBEL1842 and related *Weissella* species.

**Fig. 4:**
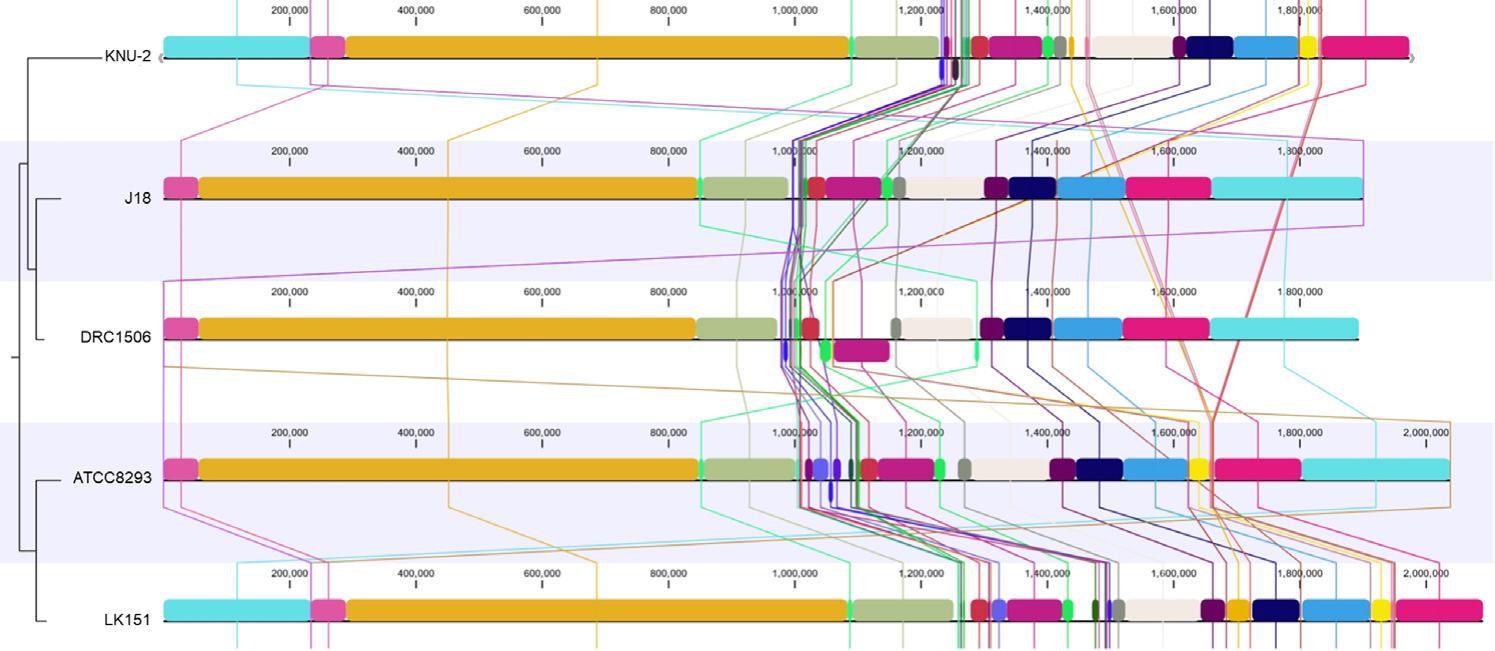
Whole-genome alignment of Leuconostoc mesenteroides strains.

**Fig. 5:**
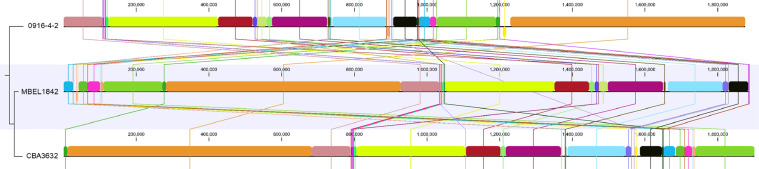
Whole-genome alignment of *Weissella hellenica* strains.

The genome sequence of *L. mesenteroides* KNU-2 was deposited in GenBank under the accession number CP089782 (https://www.ncbi.nlm.nih.gov/nuccore/CP089782) and *W. hellenica* MBEL1842 was deposited under the accession number CP086020 (https://www.ncbi.nlm.nih.gov/nuccore/CP086020). The genome sequence data provide essential information for understanding LAB in kimchi.

## 2 Experimental Design, Materials, and Methods

The *L. mesenteroides* KNU-2 (KCTC18324P) and *W. hellenica* MBEL1842 strains were cultured in MRS (de Man, Rogosa and Sharpe) broth at 37 °C for 24 h. Genomic DNA was extracted from the strains grown to an exponential phase using the G-DEX™IIc Genomic DNA Extraction kit (iNtRON, Daejeon, Korea). Whole-genome sequencing was performed using the PacBio RSII platform (Pacific Biosciences, CA, USA). If it was estimated that the total number of bases in the PacBio reads would result in less than 100 × genome coverage, additional sequencing was performed, and all the output raw data were used for assembly.

The reads of *L. mesenteroides* KNU-2 and *W. hellenica* MBEL1842 were assembled using RS HGAP (v3.0) and FALCON (v2.1.4), respectively, [1,2]. Polishing was performed using the Quiver algorithm of SMRT® analysis (v2.3.0) and the assessment was performed using BUSCO (v3.0) [11]. Gene prediction was performed using Prokka (v1.12b) to predict and annotate the open reading frame [13]. Fig. 1 shows the whole-genome sequencing steps and the parameters.

The ANI values for closely related species were calculated using the Orthologous Average Nucleotide Identity Software Tool (OAT). Whole-genome alignment of strains was visualized using the CLC Genomics Workbench 20.0.4 (CLC bio) software program.

## Ethics Statements

Not applicable.

## CRediT authorship contribution statement

**J.A. Yoon:** Methodology, Software, Data curation, Writing – original draft, Visualization. **S.Y. Kwun:** Methodology, Software, Data curation, Writing – original draft. **E.H. Park:** Writing – review & editing, Data curation. **M.D. Kim:** Conceptualization, Supervision, Funding acquisition.

## Declaration of Competing Interest

The authors declare that they have no known competing financial interests or personal relationships that could have appeared to influence the work reported in this paper.

## Data Availability

Leuconostoc mesenteroides KNU-2 Genome sequencing and assembly (Reference data) (National Center for Biotechnology Information Search database) Weissella hellenica strain:MBEL1842 Genome sequencing and assembly (Reference data) (National Center for Biotechnology Information Search database)
